# The impact of maternal obesity on in vivo uterine contractile activity during parturition in the rat

**DOI:** 10.14814/phy2.15610

**Published:** 2023-03-02

**Authors:** Ronan Muir, Raheela Khan, Anatoly Shmygol, Siobhan Quenby, Matthew Elmes

**Affiliations:** ^1^ Division of Food Nutrition and Dietetics, School of Bioscience University of Nottingham, Sutton Bonington Campus Loughborough England UK; ^2^ Graduate School of Medicine University of Nottingham, Royal Derby Hospital Derby England UK; ^3^ Department of Physiology, College of Medicine and Health Sciences United Arab Emirates University Al Ain UAE; ^4^ Biomedical Research Unit in Reproductive Health University Hospital Coventry and Warwickshire Coventry UK

**Keywords:** dysfunctional labor, dystocia, in vivo myometrial contractile activity, labor, maternal obesity, myometrial contractile function, parturition, telemetry surgery

## Abstract

Maternal obesity is associated with increased risk of prolonged and dysfunctional labor and emergency caesarean section. To elucidate the mechanisms behind the associated uterine dystocia, a translational animal model is required. Our previous work identified that exposure to a high‐fat, high‐cholesterol (HFHC) diet to induce obesity down‐regulates uterine contractile associated protein expression and causes asynchronous contractions ex vivo. This study aims to investigate the impact of maternal obesity on uterine contractile function in vivo using intrauterine telemetry surgery. Virgin female Wistar rats were fed either a control (CON, *n* = 6) or HFHC (*n* = 6) diet for 6 weeks prior to conception, and throughout pregnancy. On Day 9 of gestation, a pressure‐sensitive catheter was surgically implanted aseptically within the gravid uterus. Following 5 days recovery, intrauterine pressure (IUP) was recorded continuously until delivery of the 5th pup (Day 22). HFHC induced obesity led to a significant 1.5‐fold increase in IUP (*p* = 0.026) and fivefold increase in frequency of contractions (*p* = 0.013) relative to CON. Determination of the time of labor onset identified that HFHC rats IUP (*p* = 0.046) increased significantly 8 h prior to 5th pup delivery, which contrasts to CON with no significant increase. Myometrial contractile frequency in HFHC rats significantly increased 12 h prior to delivery of the 5th pup (*p* = 0.023) compared to only 3 h in CON, providing evidence that labor in HFHC rats was prolonged by 9 h. In conclusion, we have established a translational rat model that will allow us to unravel the mechanism behind uterine dystocia associated with maternal obesity.

## Graphical abstract

This is the first study to investigate the effect of dietary induced adiposity upon uterine contractile activity in vivo. Uterine contractile activity was measured by surgically implanting a pressure sensitive telemeter probe into the gravid uterus using aseptic techniques. Using this surgical technique, it was identified that feeding a high‐fat, high‐cholesterol diet to induce adiposity significantly prolonged labor compared to lean control fed animals.

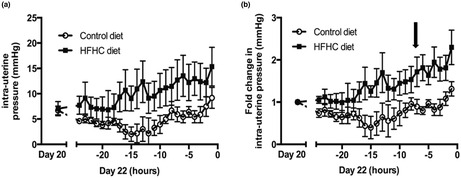

## 1 INTRODUCTION

Obesity rates in women of reproductive age are estimated to reach 50% by 2050 (Butland et al., 2007) and the National Health Service (NHS) is on the cusp of a maternity care crisis as pregnancy complications will rise significantly. Maternal obesity substantially increases the risk of caesarean delivery, postpartum hemorrhage, longer duration of hospital stay, requirement for neonatal intensive care and stillbirth (CMACE, 2010; Heselhurst et al., 2008). Emergency caesarean delivery rates are significantly increased within the obese population (Poobalan et al., 2009), because of poor uterine contractile activity, and prolonged labor (Bogaerts et al., 2013; Kominiarek et al., 2011). With a single caesarean delivery costing £1530 more than a spontaneous vaginal delivery (NICE, 2011), and the significant health risks to the parturient during and after the procedure, the need to elucidate the etiology and mechanisms behind maternal obesity induced uterine dystocia is critical.

To unravel the mechanism behind prolonged and dysfunctional labor, we established a rat model of maternal obesity. We identified that dietary high‐fat, high‐cholesterol (HFHC) induced adiposity leads to asynchronous myometrial contractions in laboring uterine strips ex vivo (Muir et al., 2016). Furthermore, the un‐coordinated and dysfunctional myometrial contractions with maternal obesity were associated with adverse effects on contractile associated protein (CAP) expression. Obese rat dams exhibited reduced protein expression of the gap junction protein connexin 43 (cx43) that synchronizes myometrial contractions during labor (Elmes et al., 2011; Muir et al., 2016) and increased expression of phosphorylated cx43 (pcx43) (phosphorylation at serine 368) compared to lean controls. Phosphorylation of cx43 (pcx43) is negatively correlated with gap junction assembly and reduces cell‐to‐cell communication (Su & Lau, 2014). Increased cx43 phosphorylation in cardiomyocytes causes gap junction disassembly and reduced contractile activity in vitro (Huang et al., 2004). These observed differences in expression of cx43 and pcx43 in the laboring uterus could explain why our maternally obese rats exhibit asynchronous contractions. Steroids hormones estrogen and progesterone regulate myometrial expression of cx43, where progesterone suppresses myometrial gap junctions during pregnancy (Garfield et al., 1980; Hendrix et al., 1992) and estrogen administration during pregnancy induces premature gap junction formation and labor in the rat (MacKenzie & Garfield, 1986). The shift in protein expression of cx43 with maternal obesity may result from higher progesterone concentrations (Elmes et al., 2011) that limits the change to a contractile uterine milieu, resulting in compromised uterine contractility.

Despite the research in this field, our current knowledge is based entirely on in vitro and ex vivo studies, no study has yet investigated the effect of maternal obesity on term‐laboring uterine contractile activity in vivo. Research published by Pierce et al. (2010) illustrated that it is possible to utilize blood pressure telemetry systems to record the change in intrauterine pressure (IUP) during term and premature labor as an indirect measure of myometrial contractile activity. The approach has the potential to determine whether maternal obesity compromises uterine function in vivo. The aim of the study was to investigate the effects of dietary HFHC induced adiposity upon uterine contractility in vivo, through surgical implantation of telemetry pressure probes to measure IUP, to test the hypothesis that maternal obesity adversely affects uterine contractile function causing prolonged and dysfunctional labor. Establishing that our rat model of maternal obesity exhibits asynchronous myometrial contractions in vivo and exhibits prolonged labor can help unravel the mechanism behind prolonged and dysfunctional labor and identify potential dietary intervention or drug therapies to improve pregnancy and labor outcomes with maternal obesity.

## 2 METHODS

### 2.1 Ethics statement

All animal work was approved by the University of Nottingham Animal Welfare Ethical Review Board (AWERB) Approval Ref No 000055 and Home Office (PPL 40/3598). All licensed procedures were carried out by licensed researchers under the Animals Scientific Procedures Act (ASPA) of 1986 within the animal facilities of the University of Nottingham. All investigators understand the ethical principles under which Physiological Reports operate, and all animal work complies with the ethics checklist.

### 2.2 Animals

Twelve weanling virgin female Wistar rats (Rattus rattus) weighing 60 g (Charles River, UK) were pair housed under normal conditions (12 h light: dark photoperiod, 21 ± 5°C room temperature, 55% ± 5% relative humidity, food and water access ad libitum) and randomly assigned to be fed either a standard control laboratory chow (CON, n = 6) (Harlan Laboratories, UK) or HFHC (*n* = 6) diet as previously published (Elmes et al., 2011). Each rat was maintained on their respective diets for 6 weeks prior to mating with stud Wistar males (Charles River, UK) and throughout pregnancy. Pregnancy was confirmed via a successful mating with a stud male, and the time of appearance of a semen plug was recorded as gestational day 0.

#### 2.2.1 Surgical procedure

At Day 9 of gestation, a chronic use TA11‐C40 pressure sensitive transmitter (Data Science International, Mn, USA) was surgically implanted into the gravid uterus using aseptic techniques. Animals were placed under general anesthesia (2% Isoflurane 95% Oxy/CO_2_), with pedal reflex and respiratory rate monitored to determine and maintain depth of anesthesia. Animals were administered preoperative buprenorphine (0.0168 mL/100 g) and meloxicam (0.004/100 g) subcutaneously to provide postoperative pain amelioration. The abdomen was prepared by shaving with clippers and swabbed with chlorhexidine and Viruscan (Cairn Technology, Sheffield, UK). Aseptically, a vertical incision was made from the xiphoid sternum to the bladder exposing the muscle layer; the muscle layer was opened following the linea alba. The gravid uterus was exteriorized to count the number of embryos within each uterine horn; the uterine horn with the greatest gravidity was selected for cannulation. Microscopically, a 2.5 mm incision was made into the uterus between the ovary and the first embryo. The pressure sensitive tip of the catheter was fed into the gravid uterus until it resided between the third and the fourth embryo. The catheter was secured using a few drops of Vet Bond (3 M, MN, USA). The cannulized horn was then interiorized back into the abdominal cavity, and the transmitter anchored to the interior of the muscle layer near the linea alba about 1 cm below the liver. The muscle layer was closed using a simple continuous suture pattern and finished with a muscle layer knot. The skin was closed using a simple continuous subcuticular stitch, with an Aberdeen knot to finish. The area around the wound was cleaned using chlorhexidine and dressed with Opsite (Opsite, London, UK). Animals were allowed to return to consciousness and provided with mash (water softened chow) for 24 h postoperatively, then maintained on their respective CON or HFHC diet for the rest of the trial. All rats recovered fully within 5 days of surgery, weight, food, and water intake daily, along with symptoms of pain or change in behavior recorded. Postoperative buprenorphine (0.0168 mL/100 g) and meloxicam(0.004/100 g) were provided subcutaneously for up to 5 days to alleviate any postoperative pain or discomfort. Following a full recovery, the transmitted data were collected every second via radio‐telemetry over an AM frequency at 500 Hz to a DSI PhysioTel receiver pad (model RPC‐1). The DSI data exchange matrix would collect and store data via DSI Dataquest A.R.T Analysis software (version 4.33); changes in the local atmospheric pressure were accounted for by the DSI Ambient Pressure reference monitor (model APR‐1). Recording of intrauterine pressure continued until term delivery of the 5th pup where rat dams were euthanized using CO_2_ asphyxiation and cervical dislocation and pups by overdose of pentobarbitone. Blood was collected via cardiac puncture into EDTA coated tubes (Sarstedt, Nümbrecht, Germany), and subsequently centrifuged at 13,000 rpm to isolate the plasma which was flash frozen and stored at −80°C. The uterus, liver, kidneys, gonadal and perineal fat depots were dissected out, weighed, and snap frozen and stored at −80°C for future research. Euthanasia of rat dams after delivery of their 5th pup was an important component of the study protocol to standardize the point at which accurate and confident comparisons could be made between the CON and HFHC rats in established labor. It also meant that if the HFHC diet did compromise uterine contractile activity and length of labor as hypothesized there was appropriate time for it to be observed experimentally.

### 2.3 Data acquisition and statistical analysis

All data were extracted using DSI Dataquest A.R.T Analysis software (version 4.33). Intrauterine pressure, integral activity, and data frequency counts calculated from extracted data using DSI Dataquest A.R.T Analysis software, Microsoft Excel (Microsoft, USA), and LabChart Reader version 8 (AD instruments, New Zealand). Frequency of contractions was determined by manually counting every single contractile event within each 1 h period from day 20 of gestation until term delivery of the 5th pup. All data were analyzed using the Statistical Package for Social Science version 21.0 (SPSS, Chicago, IL, USA) and expressed as the mean value ± SEM, with statistical significance determined by *p* ≤ 0.05. Excluding the frequency array counts for the number and strength of contractions, all data were analyzed using mixed between‐within ANOVA to determine the effect and interaction of maternal diet (CON/HFHC) and time to delivery of the 5th pup for fold‐change in intrauterine pressure and contraction frequency. As the data frequency counts were not normally distributed, they were analyzed by the nonparametric Mann–Whitney U‐test. Data frequency array counts determined the effect of maternal diet on the number of contractions within assigned pressure range bins (0–70 mmHg) at 48, 24, 12, 6, 3, and 1 h before birth of the 5th pup. All graphs were produced using GraphPad Prism version 6.0 (GraphPad, San Diego, CA, USA).

## 3 RESULTS

### 3.1 Body and fat depot weight changes

Feeding the HFHC diet 6 weeks prior to conception to induce maternal obesity significantly increased pregestational weight gain compared to controls (*p* = 0.003). Controls gained 96.02 ± 4.8 g, and HFHC gained 122.74 ± 4.9 g (see Table 1). Weight gain during pregnancy was similar between the dietary treatment groups as was the weight at the end of pregnancy. Individual perirenal and gonadal fat depot weights were higher following exposure to the HFHC diet relative to controls, and the total visceral fat mass in HFHC rats was twice the level of controls at 10.4 ± 1.82 g compared to 5.87 ± 1.01 g (*p* = 0.054, see Table 1). Importantly, exposure to the HFHC diet and the effects of intrauterine telemetry surgery did not significantly affect litter size (*p* = 0.132), pup sex ratio (*p* = 0.804), pup weights (*p* = 0.126) or lead to significant differences in the number of fetal losses (*p* = 0.365) Table 1.

**TABLE 1:** The effect of exposure to a HFHC diet to induce maternal obesity on body and fat depot weight changes.

Category	Control (*n* = 6)	HFHC (*n* = 6)	*p*‐value
Pre‐gestational weight gain (g)	96.02 ± 4.82	122.74 ± 4.91	**0.003**
Gestational weight gain (g)	131.3 ± 6.9	131.9 ± 10.82	0.963
Final weight (g)	351.82 ± 8.27	384.62 ± 16.4	0.105
Perirenal fat (g)	2.97 ± 0.53	5.3 ± 1.26	0.118
Gonadal fat (g)	2.9 ± 0.74	5.1 ± 0.91	0.09
Total visceral fat (g)	5.87 ± 1.01	10.4 ± 1.82	**0.054**
Litter size	11 ± 1.21	13.5 ± 0.92	0.132
Offspring male: female ratio	1.22 ± 0.38	1.37 ± 0.64	0.804
Average pup weight (g)	6.45 ± 0.11	6.06 ± 0.19	0.126
Fetal losses	4.33 ± 1.41	2.33 ± 0.365	0.365

*Note:* Bold indicates statistical significant value (*p* < 0.05).

### 3.2 The effect of HFHC induced obesity on contractile activity in vivo

**FIGURE 1:**
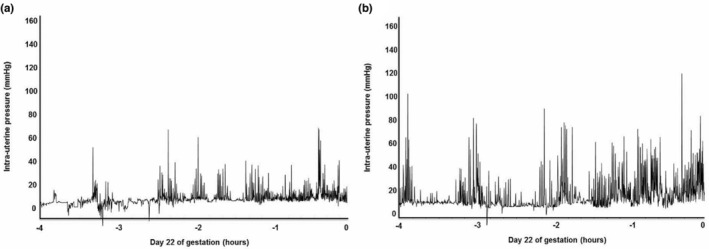
Representative traces of the change in intrauterine pressure (IUP) during the final 4 h approaching term delivery of the 5th pup at time point zero within (a) CON & (b) HFHC rats.

**FIGURE 2:**
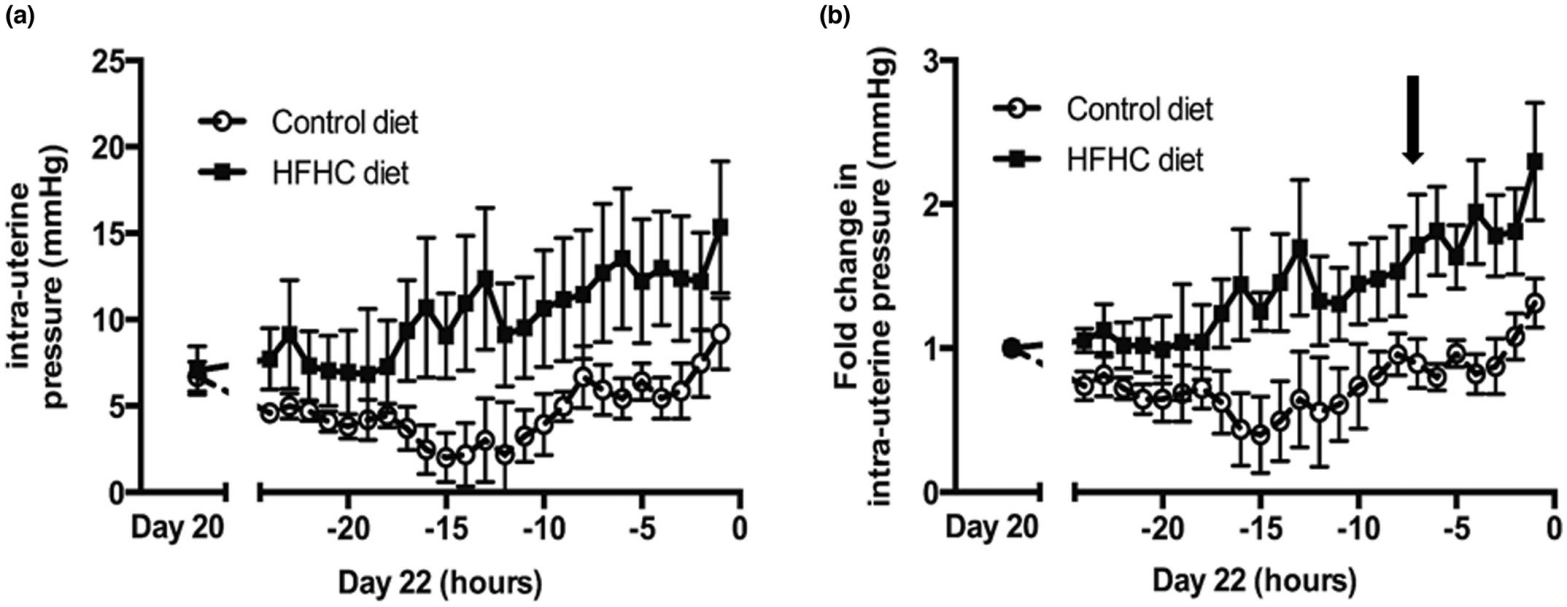
Effect of feeding a HFHC diet upon (a) intrauterine pressure & (b) fold‐change in intrauterine pressure; end of −1 h marks birth of 5th pup. Group sizes for CON (*n* = 6) and HFHC (*n* = 6) diet animals. Values are means ± SEM. Black arrow indicates the point at which there is a significant and sustained increase in intrauterine pressure above baseline.

**FIGURE 3:**
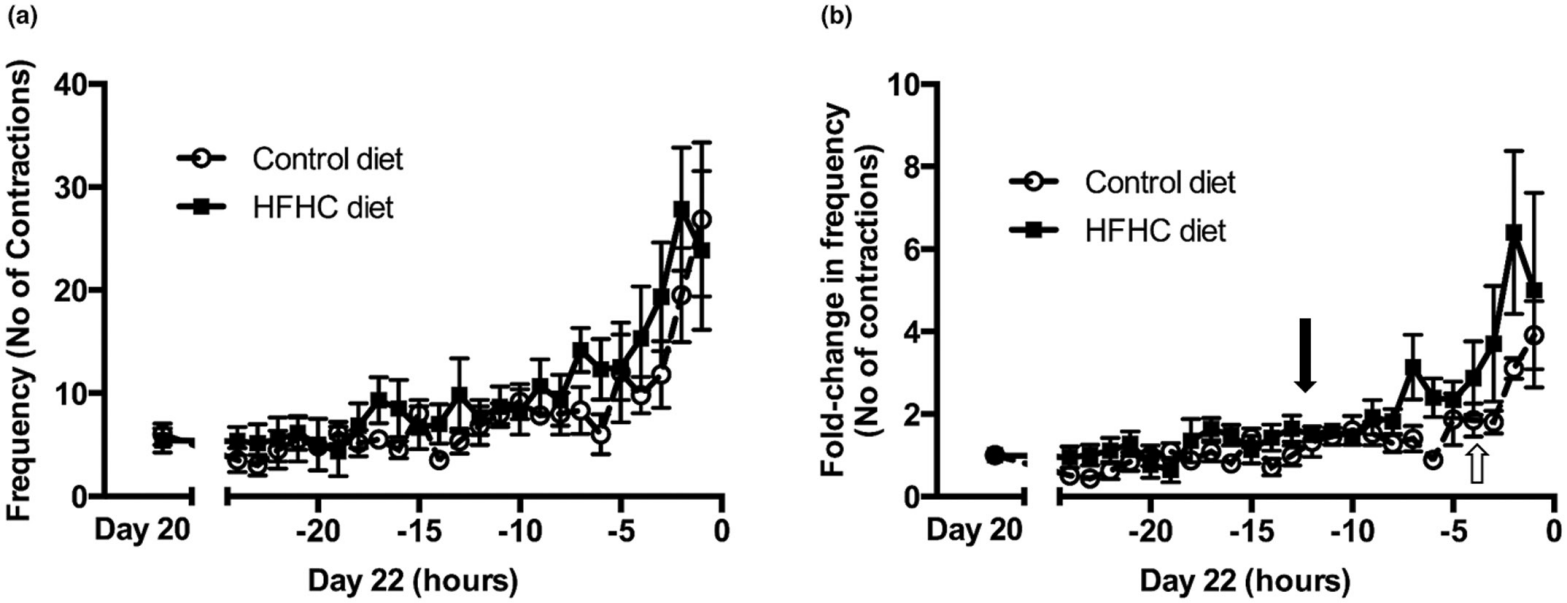
Effect of feeding a HFHC diet upon (a) contraction frequency & (b) fold‐change in contraction frequency; end of −1 h marks birth of 5th pup. Group sizes for CON (*n* = 6) and HFHC (*n* = 6) diet animals. Values are means ± SEM. Arrows; [White (CON) and black (HFHC)] identify the point at which there is a significant and sustained increase above baseline.

**FIGURE 4:**
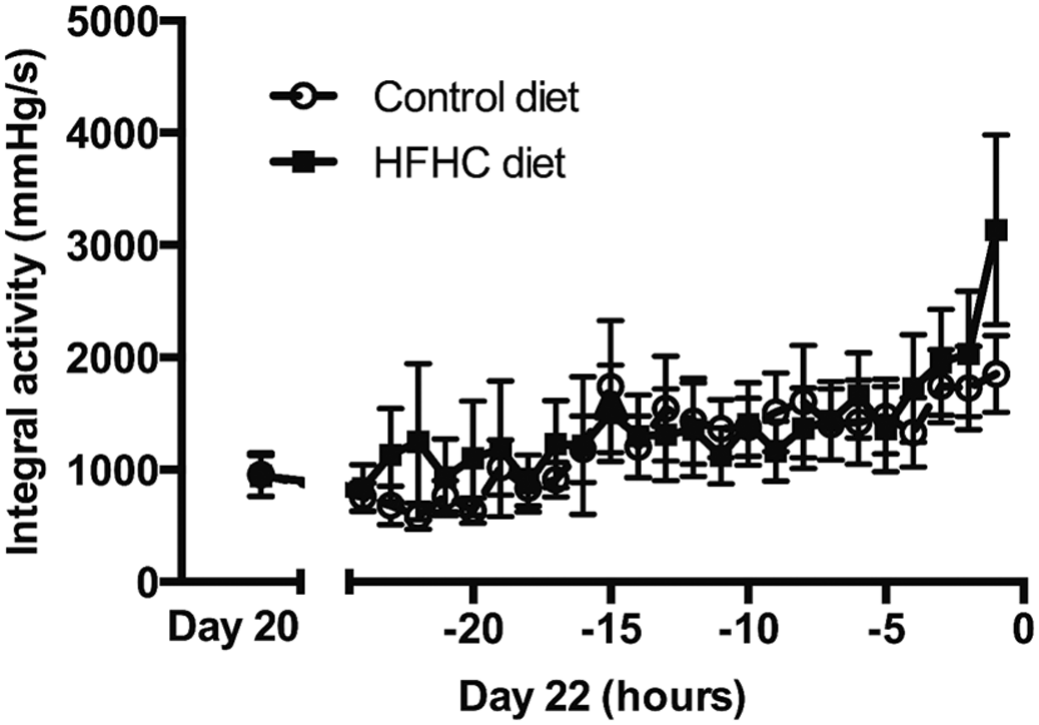
Effect of feeding a HFHC diet upon integral activity. Group sizes for CON (*n* = 6) and HFHC (*n* = 6) diet animals; end of −1 h marks birth of 5th pup. Values are means ± SEM.

Visual analysis of raw intrauterine pressure (IUP) traces from representative CON and HFHC rats shows very clearly that HFHC induced obesity leads to greater uterine contractile activity during parturition (Figure 1). More detailed analysis from Day 20 of gestation right through to delivery of the 5th pup shows that animals fed the HFHC diet displayed significant increases in both IUP (Figure 2a) and contraction frequency (Figure 3a) relative to CON animals. The HFHC fed rats exhibited a 1.5‐fold increase in IUP (*p* = 0.026) and fivefold increase in frequency of uterine contractions (*p* = 0.013) from Day 20 of gestation until delivery of the 5th pup, relative to the CON animals that showed a 0.31 and 4.0‐fold increase in IUP and contraction frequency, respectively (Figures 2b and 3b). Regardless of which diet was fed, the frequency of contractions increased significantly as labor progressed towards delivery of the 5th pup and peaked during the final hours of labor (CON (*p* = 0.012) and HFHC (*p* = 0.016)). Maternal diet did not significantly affect integral activity (*p* = 0.760). However, integral activity did increase significantly as CON (*p* = 0.036) and HFHC rats (*p* = 0.05) progressed towards delivery (Figure 4).

### 3.3 Data frequency array counts

**FIGURE 5:**
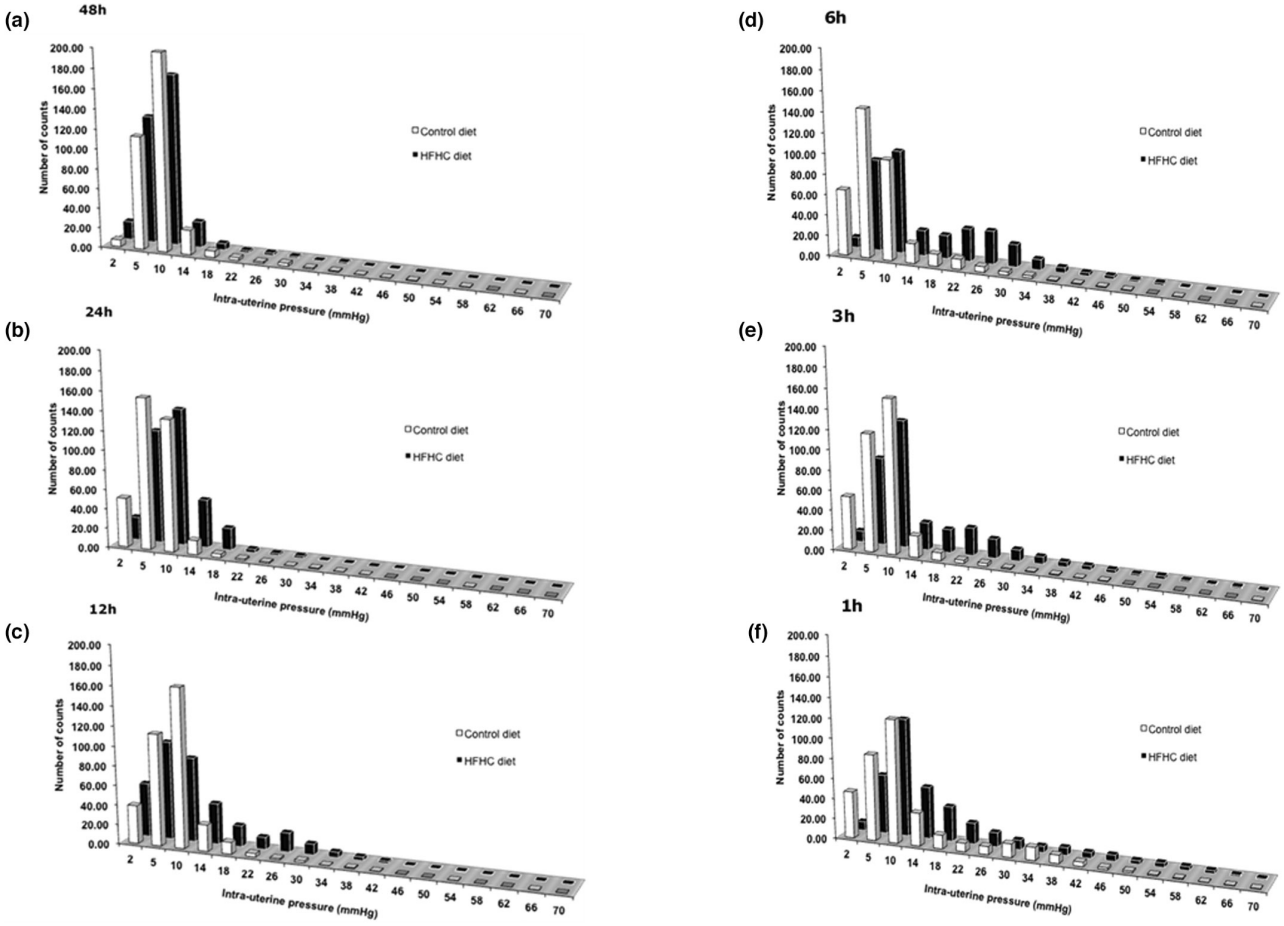
Effect of feeding a CON or HFHC diet upon data frequency array counts at (a) 48 h, (b) 24 h, (c) 12 h, (d) 6 h, (e) 3 h, and (f) 1 h prior to delivery of the 5th pup.

Data frequency array counts were carried out on IUP data collected at 48, 24, 12, 6, 3, and 1 h prior to delivery of the 5th pup and assigned into different 4 mmHg incremental bins ranging from 0 to 70 mmHg. Statistical analysis did not reveal a significant effect of diet or time on frequency counts because of high variability within the dietary groups. Despite this, the data still highlighted increased contractile activity and prolonged labor. Forty‐eight hours prior to delivery of the 5th pup, CON and HFHC animals displayed relatively low uterine contractile activity, with a high frequency of counts below an IUP of 18 mmHg (Figure 5). Twenty‐four hours later, the HFHC animals displayed an increased frequency of IUP counts above 18 mmHg relative to CON rats, this trend continued with HFHC animals displaying a rightward shift characterized by an increasing frequency of counts in higher IUP bins (>18 mmhg) at 12, 6, 3 and 1 h prior to delivery of the 5th pup. This shift continued in the HFHC rats where maximal contractions of 70 mmHg were observed 1‐h prior to delivery of the 5th pup. Unlike the HFHC animals, CON animals displayed relatively high frequency of counts below 18 mmHg throughout the same 48 h. Only at 1 h prior to delivery of the 5th pup did the frequency counts of CON rats exceed 18 mmHg where they reached the maximal mean peak of 58 mmHg.

### 3.4 The effect of HFHC induced obesity on the duration of labor

With evidence that HFHC induced obesity caused a 1.5‐fold increase in IUP and a fivefold increase in frequency of contractions, we wanted to investigate the effect of maternal diet on the timing and length of labor. Statistical analysis by one‐way ANOVA repeated measures determined the point at which IUP and contractile frequency significantly increased and remained above baseline activity in both CON and HFHC fed rats. It is evident that HFHC rats displayed a significant increase in IUP 8 h prior to delivery of the 5th pup (*p* = 0.046), which was in complete contrast to CON rats that showed no significant increase in IUP (Figure 2b). Analysis of fold change in frequency of myometrial contractions identified that HFHC rats exhibited a significant and consistent increase above baseline 12 h prior to delivery of the 5th pup (*p* = 0.023). In comparison, CON rats only experienced a significant increase in contractile frequency 3 h prior to delivery (Figure 3b) suggesting that HFHC rats labored 9 h longer than CON. These data provide evidence that animals sustained on the HFHC diet display increased contractile activity for a longer duration relative to lean CON rats. This increase in contractile activity and duration of labor is symptomatic of a prolonged duration of labor.

## 4 DISCUSSION

It is well established that obese parturients are at a significantly greater risk of exhibiting prolonged and dysfunctional labor and requiring emergency caesarean delivery (Bogaerts et al., 2013; Kominiarek et al., 2011; Poobalan et al., 2009); however, the etiology and mechanisms remain unresolved. Our previous research confirmed that exposure to a HFHC diet to induce adiposity had a significant negative effect on term‐laboring uterine contractility ex vivo, leading to un‐coordinated contractions and suppressed response to uterotonic oxytocin that is commonly used to augment labor (Muir et al., 2016). The key aim of the current study was to ascertain whether diet induced obesity also compromises in vivo uterine contractile activity during labor through surgical implantation of an intrauterine telemetry device as established by Pierce et al. (2010).

It is important to highlight that exposure to the HFHC diet to increase visceral white adipose tissue in the current study did not quite reach significance with a *p*‐value of 0.054, and differs to what we have shown previously (Muir et al., 2016). This is likely a result of the postoperative recovery period, when the rats utilized their fat as energy reserves during a period of reduced appetite and recuperation. An additional contributing factor that needs to be considered is the number of pups delivered before tissue collection, as animals in our previous trials were euthanized after delivery of the 1st pup, which may have a potential impact on their fat depot weights.

Using the novel surgical technique, we highlight a clear shift from relative uterine quiescence from gestational Day 20 to a contractile state by Day 22 of gestation. Relative to CON, the HFHC rats displayed significant increases in IUP and the number of uterine contractions. They also exhibited a higher frequency of stronger IUP data counts up to 70 mmHg from Day 20 of gestation to delivery of the 5th pup. The significant increase in uterine contractile activity provides evidence that the HFHC rats were in labor for 12 h before delivering their 5th pup compared to only 3 h in CON. This finding clearly indicates that diet induced obesity‐prolonged duration of labor and translates nicely to obese human pregnancies where prolonged labor is commonly observed (Bogaerts et al., 2013; Kominiarek et al., 2011).

Human clinical studies have established that increasing body mass is associated with prolonged duration of the first stage of labor, and in some cases a complete failure for labor to progress (Chin et al., 2012; Vahratian et al., 2005; Zhang et al., 2007).

Failure of labor to progress often stems from poor uterine contractile activity resulting in emergency caesarean delivery (Cedergren, 2009; Vahratian et al., 2005). Research has identified if labor is allowed to progress in obese parturients, all be it slowly to the 2nd stage of labor, they will produce a contraction profile similar to normal weight women and deliver vaginally (Buhimschi et al., 2004). This is similar to what was observed in the current study, where rats exposed to the HFHC diet suffered a prolonged duration of labor, but would in most instances be able to deliver to the 5th pup. Consistent with the data in the current study is the evidence from a recent clinical trial that maternal obesity causes higher basal tone of the uterine muscle and stronger contractions than leaner parturients during labor when measured by intrauterine pressure catheters, (Hautakangas et al., 2022). The suggested mechanism behind the increased basal uterine tone and stronger contractions with maternal obesity was potentially a difference in cervical dilation. Cervical tissue is connected to the muscle fibers of the uterine wall, and the status of the cervix can have an influence on the muscle tension of the uterus. A noncompliant or firmer cervix that has undergone less dilation, which may be the case with nulliparous obese pregnancies, may increase the tension of the uterus causing higher intrauterine pressure and strength of contractions (Hautakangas et al., 2022). Our previous work on the effects of diet induced obesity on ex vivo myometrial contractile activity, also identified an increased basal uterine tone compared to CON rats (Muir et al., 2016), again showing nice translation of our model to obese human pregnancy. It is pertinent to consider that the increased basal tone and contractile activity with obesity could be caused by physiological and molecular differences in the longitudinal or circular muscle layers of the myometrium. Intercellular communication through gap junctions is more intense in the circular with greater gene and protein expression of cx43 than the longitudinal layer of the bovine myometrium (Doualla‐Bell et al., 1995). Interestingly, treatment of the circular layer with ant‐estrogen inhibited expression of cx43; however, the effect was absent in the longitudinal layer, demonstrating that cx43 expression is differentially regulated in mycocytes from the circular and longitudinal layers (Doualla‐Bell et al., 1995). Future work could involve isolating the longitudinal and circular layers of the myometrium in our model to ascertain whether the increased basal tone, contractile strength and prolonged labor results from adverse changes to either muscle layer.

With a well‐established rat model of maternal obesity that exhibits prolonged labor, the potential mechanisms can now be unraveled to improve labor outcomes in obese human pregnancies. Ablation of murine uterine gene expression of cx43 by Tamoxifen has been observed to prolong labor by compromising synchronization of myometrial contractions (Döring et al., 2006). This finding matches the significant decrease in uterine expression of cx43 in the laboring uteri of HFHC rats (Elmes et al., 2011; Muir et al., 2016) that also exhibit asynchronous myometrial contractions ex vivo (Muir et al., 2016). It would be interesting to use the same intrauterine pressure approach to investigate whether inhibition of cx43 causes the same contractile phenotype as observed in model of maternal obesity. High circulating plasma concentrations of progesterone impede activation of uterine contractile activity by decreasing uterine expression of cx43, leading to poor synchronization of contractions during labor (Challis et al., 2000). Increased progesterone concentrations also negatively impact expression of key CAP's within the uterus including Cav‐1, OXTR and COX‐2 (Elmes et al., 2011) and limit expression of ion channels (Shmygol et al., 2007; Smith et al., 2005; Zhang et al., 2007), receptors (Gimpl & Fahrenholz, 2000, 2002; Klein et al., 1995), and uterotonin signaling (Myatt & Lye, 2004). Exposure to a HFHC diet significantly increases plasma cholesterol concentrations, that can inhibit myometrial contractile strength directly (Smith et al., 2005) or increase membrane fluidity compromising integral protein expression and down‐stream contractile signaling mechanisms (Gimpl & Fahrenholz, 2000; Pucadyil & Chattopadhyay, 2006; Shmygol et al., 2007). Disruption of the human ether‐a‐go‐go related gene (hERG) ion channel for example increases the duration of individual uterine contractions, promoting poor uterine contractility (Parkington et al., 2014). A further potential mechanism that could be detrimental to uterine contractile activity during parturition include increased synthesis of utero‐relaxant adipokines such as leptin by excess visceral white adipose tissue (Hehir & Morrison, 2012; Moynihan et al., 2006; Mumtaz et al., 2015). Despite all this knowledge, further research is required to elucidate the mechanisms behind uterine dystocia associated with maternal obesity. The rat model of maternal obesity presented in the current study would help to achieve this.

In summary, surgical implantation of the pressure sensitive telemetry system is a novel approach to determine whether chronic exposure to a HFHC diet had a negative effect upon IUP and uterine contractility during term labor in vivo. This study provides clear evidence that HFHC induced obesity‐prolonged labor duration significantly by 9 h compared to lean CON rats. These findings translate nicely to obese human pregnancies meeting the aims and hypothesis of the study. The data presented highlights the versatility of this dietary animal model and surgical procedure in investigating the mechanism(s) behind prolonged and dysfunctional labor associated with maternal obesity. The methods and model presented here could be applied to investigate any number of hypotheses governing uterine dystocia or the multifactorial interaction of the body systems to trigger and sustain parturition with maternal obesity.

## 5 CONCLUSION

This is the first known study to investigate the effect of exposure to a HFHC diet to induce maternal obesity on uterine contractility in vivo, by surgically implanting a pressure‐sensitive catheter into the gravid uterus. Through this novel approach, we identified that exposure to the HFHC diet leads to a 9‐h prolongation of labor relative to lean CON rats. This study in combination with our previous published research now provides an established translational rat model of maternal obesity that can be utilized to investigate and identify the mechanism(s) through which maternal obesity compromises uterine contractility during labor using both ex vivo and now in vivo methods.

## AUTHOR CONTRIBUTIONS

Study design, management, and theater assistance was performed by Matthew Elmes. Daily running of the animal trial and all the surgical procedures carried out by Ronan Muir. The manuscript was written and reviewed by Ronan Muir and Matthew Elmes. The manuscript was reviewed by Ronan Muir, Raheela Khan, Anatoly Shmygol, Siobhan Quenby, and Matthew Elmes.

## FUNDING INFORMATION

This work was funded by the University of Nottingham BBSRC‐DTP PhD studentship, University Hospitals Coventry & Warwickshire NHS Trust studentship [grant number BB/J014508/1] and Rosetrees Trust [grant number M449].

## CONFLICT OF INTEREST STATEMENT

The authors have no conflicts of interest to report.

## AUTHOR'S TRANSLATIONAL PERSPECTIVE

This study and previous research by our group have highlighted the clear efficacy and translatability of this dietary animal model in the study of maternal obesity associated uterine dystocia. This dietary animal model could be used to investigate and identify the aberrant mechanism by which obesity compromises uterine contractility during parturition. This could lead to the development and understanding of pharmacological or dietary interventions to help curb the significantly high rates of caesarean delivery resulting from prolonged and dysfunctional labor associated with maternal obesity.
